# Cavity Effects and Prediction in the Vibration of Large-Section Rectangular Coal Roadways Induced by Deep-Hole Bench Blasting in Open-Pit Mines

**DOI:** 10.3390/s25113393

**Published:** 2025-05-28

**Authors:** Anjun Jiang, Honglu Fei, Yu Yan, Runcai Bai, Shijie Bao

**Affiliations:** 1 Institute of Blasting Technology, Liaoning Technical University, Fuxin 123000, China; jianganjun_cn@126.com (A.J.); hityanyu@163.com (Y.Y.); 15041814194@163.com (S.B.); 2 School of Civil Engineering, Liaoning Technical University, Fuxin 123000, China; 3 School of Mining, Liaoning Technical University, Fuxin 123000, China; bairuncai@126.com

**Keywords:** open-pit bench blasting, blasting seismic wave, rectangular roadway, cavity effect, peak particle velocity, velocity prediction

## Abstract

The dynamic response law of the vibration cavity effect in the adjacent large-section rectangular coal roadways induced by deep-hole bench blasting vibrations was deeply revealed, and the prediction accuracy of the PPV in the coal roadway was improved. The vibration equations of the coal roadway were derived based on the stress wave propagation theory and the wave-front momentum conservation theorem. Based on coal roadway vibration monitoring data and numerical simulations, the cavity effect and vibration response characteristics of the coal roadway induced by deep-hole bench blasting under varying blast source distances and relative angle conditions were analyzed. A predictive model for PPV of rectangular coal roadway surrounding rock, incorporating the relative angle as one of the key influencing factors, was developed. The results showed that the presence of cavities and changes in the relative angle enhance the asymmetry of the dynamic response of blasting stress waves near the free surfaces of the surrounding rock on each side of the coal roadway, resulting in significant differences. Moreover, as the blasting distance decreases, the cavity effect tends to promote greater PPV differences on each side of the coal roadway. The prediction model exhibited improved accuracy by about 15.6% compared to the traditional Sadovski equation for the face-blasting side of the coal roadway. It demonstrates better adaptability and predictive capability. This research provides a theoretical basis for the dynamic response of adjacent large-section rectangular coal roadways and for preventing dynamic instability failures in open-pit mining.

## 1. Introduction

Open-pit bench blasting is widely employed in mining engineering due to its cost effectiveness and high efficiency. However, drilling and blasting inevitably generate harmful effects [1,2], and the effect of blast-induced vibration on the stability of buildings (structures) within a specific range is the most prominent [3,4]. In particular, when the open-pit mining areas are adjacent to underground mining zones, the effects of blast-induced vibration pose a severe threat to the stability and safety of adjacent underground large-section rectangular coal roadways, such as wall caving, crack development in the surrounding rock, roof collapse, etc. Therefore, the dynamic response of large-section rectangular coal roadway surrounding rock under the effect of blast-induced vibration should be investigated in open-pit mining, and an accurate model for predicting the peak particle velocity (PPV) of the surrounding rock should be established. It is of great engineering significance for preventing damage to large-section rectangular coal roadways caused by intense vibrations resulting from large-charge, deep-hole bench blasting in open-pit mines, as well as ensuring their structural stability.

The PPV is a primary metric for assessing the strength of blast-induced vibrations. It is a key parameter in blast vibration safety standards [5,6]. Therefore, it is often used as a control criterion for blast vibration.

In terms of open-pit blasting, Duvall and Fogelson [7] investigated the attenuation of blasting seismic waves and established seismic standards for various buildings. The USBM investigators Ambraseys and Hendron [8] advised the actual distance to be divided by the cube root of the charge weight. Bilgin et al. [9] proposed an equation by adding a burden parameter to classical PPV prediction models. Rai and Singh [10] proposed a PPV prediction equation considering an inelastic attenuation factor and analyzed its calculation results in comparison with other vibration prediction equations for the same dataset. The analysis shows that this prediction formula can be effectively used for calculating the safe charge per delay more precisely. Ak and Konuk [11] investigated the impacts of discontinuity frequency on the propagation of blast-induced vibration in bench blasting and established the relationship between the scaled distance and discontinuity frequency with the PPV. Y. Yan et al. [12] summarized the influential parameters on blast-induced ground vibrations and evaluated the free face, maximum charge per delay, distance from the blasting source to the monitored point, and geological conditions, confirming their influence in predicting the PPV. Khandelwal et al. [13] analyzed various blasting design parameters on predicting the PPV and proposed a new empirical equations based on dimensional analysis. It comprehensively reflected the influence of parameters such as the Young’s modulus of rock mass, powder factor, depth and hole diameter, spacing, burden, and charge length on the attenuation law of blast-induced vibration. W. M. Yan et al. [14] found that the influential parameters and empirical equations for predicting the PPV were uncertain and proposed using the Bayesian technique and Monte Carlo simulation to predict the PPV. This method could offer practicing engineers confidence intervals when the equation is used to estimate a blast-induced vibration. Based on the scaled distance concept used for inelastic attenuation factor, Simangunsong and Wahyudi [15] analyzed the effect of a bedding plane on the prediction of blast-induced ground vibration in open-pit coal mines. Based on the analysis results, the relationship between the incident angle, the number of coal layers, and the PPV was established. Yilmaz [16] proposed a new equation for predicting the PPV, for which the coefficient of determination of regression results was high, indicating the correlation between the scaled distance and the PPV with inelastic attenuation. Kumar et al. [17] proposed an empirical equation for predicting the PPV by considering the effects of rock mechanical and geological properties, like the unit weight, rock quality designation (RQD), geological strength index (GSI), and uniaxial compressive strength (UCS). Chen et al. [18] investigated the influence of the cylindrical charge length on blast-induced vibration based on elastic–plastic theory and provided a new equation for predicting the PPV considering the characteristics of cylindrical charge length. Murmu et al. [19] proposed two empirical equations, one considering the overburden and the other without it. Compared with the regression results of two empirical equations, the results showed that overburden did not significantly affect PPV. Yin et al. [20] investigated different rock masses on the attenuation characteristics of blast-induced vibration, The results indicated that the development degree of rock mass joints affected the attenuation rate of the PPV. It indicated that the more joints there are, the higher the attenuation rate of the PPV. A modified equation for predicting PPV was developed by considering the joint number in the rock mass. Deng et al. [21] derived the empirical equation for predicting PPV, which was modified for multi-hole blasting based on stress wave propagation theory. The proposed model could adequately reflect the propagation of PPV under multi-hole blasting.

In terms of underground blasting, Nan Jiang et al. [22] investigated the blast-induced vibration attenuation of the slope subjected to underground mining and analyzed the distribution characteristics of PPV. Meanwhile, a PPV prediction model suitable for open-pit mine slopes subjected to underground mining considering the mining depth and elevation was proposed. Nan Jiang et al. [23] investigated the impact of subway excavation on adjacent buried gas pipelines, and a mathematical model was established to describe the attenuation of the PPV with respect to the depth of tunnel blasting. Wang et al. [24], based on the diffraction and reflection amplification effects of blast-induced vibration around tunnels, calculated the possible minimum diffraction distance of blast-induced vibration around the rock tunnels and improved the scaled distance (SD) of the empirical equations to predict the PPV on adjacent tunnel sections. This equation efficiently enhanced the accuracy of predicting the PPV. W. Li et al. [25] compared several empirical equations for predicting the PPV of the adjacent tunnel surrounding rock based on the rock strength index (GSI). The improved equations could be adopted to predict the PPV for complex geological conditions.

In the field of machine learning and artificial intelligence, and with its rapid development [26,27], these methods are widely used in predicting blast-induced vibration and show excellent applicability.

Many research studies have been carried out on the effect of open-pit bench blasting and the corresponding influence of blast-induced vibration on the dynamic response of underground roadways. Litwiniszyn J [28] carried out a theoretical investigation into dynamic instability in underground roadways caused by blast-induced stress waves. It was found that a rarefaction stress wave and a jump-like change in the state of stress in the surrounding rock are generated when the surrounding rock reaches the instability condition. Within the interval of the jump-like change in stress, the skeleton of the surrounding rock is destroyed, and an outburst is initiated. Singh et al. [29,30] analyzed the dynamic response of blast-induced vibration on the top plate, pillar, and bottom plate of an adjacent roadway in open-pit blasting and determined the safety thresholds for the vibration of the underground roadway surrounding rocks. Duan et al. [31] evaluated the stability of open-pit bench blast seismic waves on the surrounding rocks and ancillary structures of underground roadways. However, the influence of the roadway cavity and the dynamics response of the relative position on the evolution of the surrounding rock and the accuracy of predicting the PPV have not been considered in previous studies.

Although the research on blast-induced vibration PPV control and prediction has been fully developed and applied, the influence of the differences in reflection and stacking effects of blasting stress waves on the coal roadway surrounding rock on each side (i.e., “the cavity effect”), as well as the relative position of the blasting-induced dynamic disturbance, on stress evolution and the accuracy of PPV prediction in large-section rectangular coal roadway surrounding rock has not yet been thoroughly investigated. Therefore, in this paper, the vibration response of roadway surrounding rock was carried out using stress wave propagation theory and conservation laws of wave-front momentum. Then, the dynamic response of the large-section rectangular coal roadway surrounding rock was analyzed under varying blast source distances and relative angle (i.e., θ, the relative angle between the position of the open-pit bench blasting with the underground coal roadway) conditions using finite element (FE) analysis. Finally, based on the analysis of the cavity effect in the vibration response of large-section coal roadway surrounding rock, a PPV prediction model incorporating the relative angle (θ) as one of the key influencing factors was developed, which aimed to significantly improve adaptability and prediction accuracy, providing a more precise tool for predicting the vibration response of the large-section coal roadway surrounding rock. This research provides the theory for the precise prevention of underground large-section coal roadway dynamic instability failures under vibrations due to open-pit bench blasting.

## 2. Vibrating Equations for the Adjacent Roadway in Open-Pit Bench Blasting

**Figure 1:**
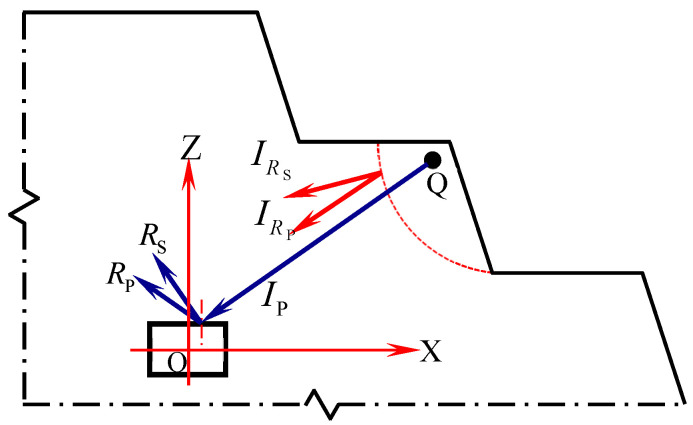
Reflection of the blast-induced stress wave on the free face of the roadway surrounding rock.

The open-pit bench blasting load enhances the dynamic response of the underground roadway surrounding rock, and the stress in the surrounding rock is redistributed under the action of blast-induced and surface reflection stress waves. When stress intensity is greater than the dynamic strength threshold of the rock mass, it promotes crack propagation and expansion in the surrounding rock, deteriorating the properties and reducing the bearing capacity of the surrounding rock, thereby inducing sudden instability in the surrounding rock. In open-pit bench blasting, underground roadways are mainly affected by blast-induced stress waves and reflected stress waves from the surface. As shown in Figure 1, after the blast-induced stress wave is reflected on the surface, it generates reflected P-waves and S-waves. Meanwhile, the incident wave generates reflected P-waves and S-waves on the free surface of the roadway surrounding rock.

The calculations were further simplified by adopting the following steps:

(1) The cylindrical charge was simplified to spherical charge, while only P-waves with a constant excitation frequency were considered; this assumption was suitable for the preliminary approximate analysis, but it may ignore the different propagation characteristics of the cylindrical charge in the axial and radial directions.
(2) The rock mass was assumed to be a homogeneous and isotropic linear elastomer.
(3) The compressive stress was defined to be positive.

In the analysis, the case of blast-induced stress waves and surface-reflected stress waves acting on the free face of the roadway surrounding rock was considered a special case of stress wave propagation in joints [32], i.e., the rock on the other side of the joints was replaced by air (wave impedance is 0). This was based on the theory of stress wave propagation and the theorem of momentum conservation in front of the wave, combined with the time-domain recursive method [32,33,34], to carry out the derivation of vibrating equations for the surrounding rock of cavity roadway under the action of blasting stress waves and surface-reflected stress waves.

### 2.1. Interaction of the Blast Stress Wave with the Surface

**Figure 2:**
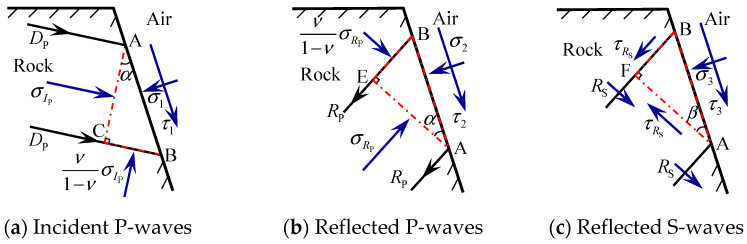
The incidence and reflection of stress waves from a tiny element on the free face.

As illustrated in Figure 2a, when an incident P-wave propagates to a free face, a tiny element ABC consists of AB, AC, and BC, where AB is the free face of the surface, BC and AC are the wave beam and the wave front of the P-wave, respectively, and is α the angle of incidence. The tiny element of the reflected P-wave interacting with the rock is ABE, BE is the wave front of the reflected P-wave, and AE is the wave beam of the reflected P-wave. According to Snell’s law [32], the incidence and the reflection angles of the P-wave are equal, so the angle of the reflected P-wave is also α, as shown in Figure 2b. Similarly, the tiny element of the reflected S-wave interacting with the rock is ABF, and BF, AF, and β are the wave front, wave beam, and the angle of the reflected S-wave, respectively, as shown in Figure 2c.

Approximating the current model as a one-dimensional strain plane wave problem, assuming that the transverse dimension of the free surface is infinitely large, the plane wave can be regarded as a one-dimensional strain wave for analysis, and combined with one-dimensional strain elastic wave theory, the stress on the BC is determined to be equal to $\sigma_{I_{P}}\cdot \nu /\left(1-\nu \right)$ [33,35,36], where $\sigma_{I_{P}}$ is the normal stress of the wave front of the incident P-wave, and ν denotes the Poisson’s ratio of the rock.

Without considering gravity, the stress for the tiny elements ABC of the surface is shown in Figure 2a, where $\sigma_{1}$ and $\tau_{1}$ are the direct and shear stresses of the rock on the left side of the free face, respectively. From the force equilibrium condition of tiny elements, the stresses in the vertical direction and radial direction of the free surface AB on the element ABC must satisfy Equations (1) and (2) [34].

In the vertical direction of the free surface AB:

(1) $$\sigma_{1}-\sigma_{I_{P}}\cos^{2}\alpha -\frac{\nu }{1-\nu }\sigma_{I_{P}}\sin^{2}\alpha =0$$

In the radial direction of the free surface AB:

(2) $$\tau_{1}-\sigma_{I_{P}}\sin\alpha \cos\alpha +\frac{\nu }{1-\nu }\sigma_{I_{P}}\cos\alpha \sin\alpha =0$$

According to the Snell’s law [34]:

(3) $$\frac{\sin\beta }{\sin\alpha }=\frac{c_{S}}{c_{P}}=\sqrt{\frac{1-2\nu }{2(1-\nu )}}$$

where $c_{P}$ and $c_{S}$ are the velocities of the P-wave and S-wave in intact rock, respectively.

The expression for ν considering sinα and sinβ can be obtained and substituted into Equations (1) and (2) and can be expressed as Equation (4).

(4) $$\sigma_{1}=\sigma_{I_{P}}\cos2\beta ,\;\;\tau_{1}=\sigma_{I_{P}}\sin2\beta \tan\beta \cot\alpha$$

Similarly, under the influence of reflected P-waves and reflected S-waves, the stresses $\sigma_{i}$ and $\tau_{i}$ (i=2~3) on the tiny elements ABE and ABF on the free face can be expressed as Equations (5) and (6).

(5) $$\sigma_{2}=\sigma_{R_{P}}\cos2\beta ,\;\tau_{2}=-\sigma_{R_{P}}\sin2\beta \tan\beta \cot\alpha$$

(6) $$\sigma_{3}=-\tau_{R_{S}}\sin2\beta ,\;\tau_{3}=-\tau_{R_{S}}\cos2\beta$$

Then, the total stress on the free face can be expressed as Equations (7) and (8).

(7) $$\begin{array}{ll} \sigma & =\sigma_{1}+\sigma_{2}+\sigma_{3}\\ & =\sigma_{I_{P}}\cos2\beta +\sigma_{R_{P}}\cos2\beta -\tau_{R_{S}}\sin2\beta \end{array}$$

(8) $$\begin{array}{ll} \tau & =\tau_{1}+\tau_{2}+\tau_{3}\\ & =\sigma_{I_{P}}\sin2\beta \tan\beta \cot\alpha -\sigma_{R_{P}}\sin2\beta \tan\beta \cot\alpha -\tau_{R_{S}}\cos2\beta \end{array}$$

On the boundary of the free surface, the stresses satisfied the conditions of σ=0 and τ=0. Based on Equations (7) and (8), the reflection stresses of the surface free boundary can be expressed as Equations (9)–(11).

(9) $$\left[\begin{array}{l} \sigma_{R_{P}}\\ \tau_{R_{S}} \end{array}\right]=-B^{-1}A\cdot \sigma_{I_{P}}$$

(10) $$A=\left[\begin{array}{c} \cos2\beta \\ \sin2\beta \tan\beta \cot\alpha \end{array}\right]$$

(11) $$B\left.=\left[\begin{array}{cc} \cos2\beta & \sin2\beta \\ -\sin2\beta \tan\beta \cot\alpha & -\cos2\beta \end{array}\right.\right]$$

### 2.2. Interaction of Blasting Stress Wave and Surface-Reflected Wave with Roadway Surrounding Rock

**Figure 3:**
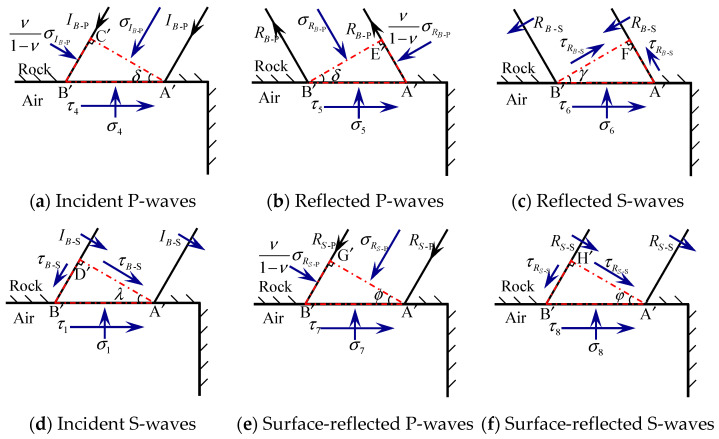
Incidence and reflection of stress waves from tiny elements on the surrounding rock of the roadway.

Similar to stress waves interacting with the ground surface, the force state on tiny elements of the roadway surrounding rock acted by the incident P-wave and the surface-reflected wave is shown in Figure 3, respectively.

In Figure 3, $\sigma_{I_{B\text{-}P}}$ and $\sigma_{R_{B\text{-}P}}$ denote the normal stresses of the incident P-wave and reflected P-waves on their wave fronts under blasting stress, respectively; $\tau_{R_{B\text{-}S}}$ denotes the shear stresses of the reflected S-waves on the wave fronts under blasting stress. $\sigma_{R_{S\text{-}P}}$ and $\tau_{R_{S\text{-}S}}$ denote the normal stress of P-waves and the shear stress of S-waves reflected from the surface, respectively. θ is the angle of incidence.

For the elements $\;A_{\prime }\;B_{\prime }\;C_{\prime }$, $\;A_{\prime }\;B_{\prime }\;E$, $\;A_{\prime }\;B_{\prime }\;F_{\prime }$, $\;A_{\prime }\;B_{\prime }\;G_{\prime }$, and $\;A_{\prime }\;B_{\prime }\;H_{\prime }$ on the free face of the roadway surrounding rock in Figure 3, the stresses $\sigma_{i}$ and $\tau_{i}$ (i=4~8) can be expressed as Equations (12)–(16):

(12) $$\sigma_{4}=\sigma_{I_{B\text{-}P}}\cos2\gamma ,\;\;\tau_{4}=\sigma_{I_{B\text{-}P}}\sin2\gamma \tan\gamma \cot\delta$$

(13) $$\sigma_{5}=\sigma_{R_{B\text{-}P}}\cos2\gamma ,\;\tau_{5}=-\sigma_{R_{B\text{-}P}}\sin2\gamma \tan\gamma \cot\delta$$

(14) $$\sigma_{6}=-\tau_{R_{B\text{-}S}}\sin2\gamma ,\;\tau_{6}=-\tau_{R_{B\text{-}S}}\cos2\gamma$$

(15) $$\sigma_{7}=\sigma_{R_{S\text{-}P}}\cos2\varphi ,\;\;\tau_{7}=\sigma_{R_{S\text{-}P}}\sin2\varphi \tan\varphi \cot\phi$$

(16) $$\sigma_{8}=-\tau_{R_{S\text{-}S}}\sin2\varphi ,\;\tau_{8}=-\tau_{R_{S\text{-}S}}\cos2\varphi$$

where δ is the angle of the incident P-wave by the blasting stress, which is equal to the angle of the reflected P-wave; γ is the angle of the reflected S-wave by the blasting stress; ϕ is the angle of incident P-waves reflected from the surface; and φ is the angle of S-waves reflected from the surface.

According to the law of conservation of momentum in the wave front, $\sigma_{I_{B\text{-}P}}=z_{P}v_{I_{B\text{-}P}}$, $\sigma_{R_{B\text{-}P}}=z_{P}v_{R_{B\text{-}P}}$, $\tau_{R_{B\text{-}S}}=z_{S}v_{R_{B\text{-}S}}$, $\sigma_{R_{S\text{-}P}}=z_{P}v_{R_{S\text{-}P}}$, and $\tau_{R_{S\text{-}S}}=z_{S}v_{R_{S\text{-}S}}$, where $v_{I_{B\text{-}P}}$ and $v_{R_{B\text{-}P}}$ are the particle velocities of the incident and reflected P-waves of blasting stress, respectively; $v_{R_{S\text{-}P}}$ is the particle velocity of the reflected P-waves of surface-reflected waves; and $v_{R_{B\text{-}S}}$ and $v_{R_{S\text{-}S}}$ are the particle velocities of the reflected S-waves of blasting stress waves and surface reflection waves, respectively. Hence, the correlation between the stresses on the free face of the roadway surrounding rock and the particle velocities was established. We define $z_{P}=\rho c_{P}$ and $z_{S}=\rho c_{S}$, where ρ is the density of the intact rock; the meanings of $c_{P}$ and $c_{S}$ are the same as above.

According to the law of momentum conservation at the wave front, the time interval between the incident stress wave and the reflected stress wave is negligible. As a result, both the incident and reflected stress waves act simultaneously on the roadway surrounding rock. Thus, the sum of stresses at the units on the free face of the roadway surrounding rock can be expressed as Equations (17) and (18).

(17) $$\begin{array}{ll} \sigma & =\sigma_{4}+\sigma_{5}+\sigma_{6}+\sigma_{7}+\sigma_{8}\\ & =z_{P}v_{I_{B\text{-}P}}\cos2\gamma +z_{P}v_{R_{B\text{-}P}}\cos2\gamma -z_{S}v_{R_{B\text{-}S}}\sin2\gamma +z_{P}v_{R_{S\text{-}P}}\cos2\varphi -z_{S}v_{R_{S\text{-}S}}\sin2\varphi \end{array}$$

(18) $$\begin{array}{ll} \tau & =\tau_{4}+\tau_{5}+\tau_{6}+\tau_{7}+\tau_{8}\\ & =z_{p}v_{I_{B\text{-}P}}\sin2\gamma \tan\gamma \cot\delta -z_{p}v_{R_{B\text{-}P}}\sin2\gamma \tan\gamma \cot\delta -z_{s}v_{R_{B\text{-}S}}\cos2\gamma \\ & \;\;\;\;-z_{p}v_{R_{S\text{-}P}}\sin2\varphi \tan\varphi \cot\phi -z_{s}v_{R_{S\text{-}S}}\cos2\varphi \end{array}$$

On the free face of the roadway surrounding rock, the stresses still satisfy σ=0 and τ=0. Equations (17) and (18) can be expressed in matrix form as Equation (19), whereas the individual matrices are given by Equations (20)–(22).

(19) $$\left.\left[\begin{array}{c} v_{R_{B\text{-}P}}\\ v_{R_{B\text{-}S}} \end{array}\right.\right]=-D^{-1}C\cdot v_{I_{B\text{-}P}}-D^{-1}E\left.\left[\begin{array}{c} v_{R_{S\text{-}P}}\\ v_{R_{S\text{-}S}} \end{array}\right.\right]$$

(20) $$C=\left[\begin{array}{c} z_{P}\cos2\gamma \\ z_{P}\sin2\gamma \tan\gamma \cot\delta \end{array}\right]$$

(21) $$D\left.=\left[\begin{array}{cc} z_{P}\cos2\gamma & -z_{S}\sin2\gamma \\ -z_{P}\sin2\gamma \tan\gamma \cot\delta & -z_{S}\cos2\gamma \end{array}\right.\right]$$

(22) $$E\left.=\left[\begin{array}{cc} z_{P}\cos2\varphi & -z_{S}\sin2\varphi \\ -z_{P}\sin2\varphi \tan\varphi \cot\phi & -z_{S}\cos2\varphi \end{array}\right.\right]$$

The normal components of the velocities on the free face of the roadway surrounding rock can be expressed as Equation (23).

(23) $${\bar{v}}_{n}=\cos\delta \cdot v_{B_{P}}-\cos\delta \cdot v_{R_{B\text{-}P}}+\sin\gamma \cdot v_{R_{B\text{-}S}}-\cos\phi \cdot v_{R_{S\text{-}P}}+\sin\varphi \cdot v_{R_{S\text{-}S}}$$

The tangential components of the velocities on the free face of the roadway surrounding rock can be expressed as Equation (24).

(24) $${\bar{v}}_{\tau }=\sin\delta \cdot v_{B_{P}}+\sin\delta \cdot v_{R_{B\text{-}P}}+\cos\gamma \cdot v_{R_{B\text{-}S}}+\sin\delta \cdot v_{R_{S\text{-}P}}+\cos\gamma \cdot v_{R_{S\text{-}S}}$$

Equations (23) and (24) can be expressed in matrix form, as given in Equations (25)–(29).

(25) $$\left[\begin{array}{c} {\bar{v}}_{n}(t)\\ {\bar{v}}_{\tau }(t) \end{array}\right]=A_{P}v_{I_{B\text{-}P}}+B_{P}\left[\begin{array}{l} v_{R_{B\text{-}P}}(t)\\ v_{R_{B\text{-}S}}(t) \end{array}\right]+C_{P}\left[\begin{array}{l} v_{R_{S\text{-}P}}(t)\\ v_{R_{S\text{-}S}}(t) \end{array}\right]$$

(26) $$\left[\begin{array}{c} {\bar{v}}_{n}(t)\\ {\bar{v}}_{\tau }(t) \end{array}\right]=(A_{P}-B_{P}B_{P}^{-1}A)\cdot v_{I_{B\text{-}P}}(t)+C_{P}\left[\begin{array}{l} v_{R_{S\text{-}P}}(t)\\ v_{R_{S\text{-}S}}(t) \end{array}\right]$$

(27) $$\left.A_{p}=\left[\begin{array}{c} \cos\delta \\ \sin\delta \end{array}\right.\right]$$

(28) $$B_{p}=\left[\begin{array}{cc} -\cos\delta & \sin\gamma \\ \sin\delta & \cos\gamma \end{array}\right]$$

(29) $$C_{p}=\left[\begin{array}{cc} -\cos\phi & \sin\varphi \\ \sin\phi & \cos\varphi \end{array}\right]$$

Equation (26) can be expressed as the vibration equation on the roadway surrounding rock induced by open-pit bench blasting. It is concluded that the particle velocity on the roadway surrounding rock is related to the surrounding rock density, the wave velocity of P- and S-waves, and the angles of incidence and reflection. It confirmed that for the same geological conditions, rock density, wave velocity, and distance between the blasting area and the roadway, the incidence angle of the stress wave determines the characteristics of the dynamic response at different locations of the roadway surrounding rock, leading to disparities in the particle velocity at various locations of the roadway surrounding rock under the bench blasting.

## 3. Dynamic Response of the Roadway Surrounding Rock

In Section 2, the vibration equation of the roadway surrounding rock under the bench blasting was derived. However, it cannot reflect the influence of the relative angle (θ) and the cavity effect on the dynamic response of the roadway surrounding rock. Therefore, numerical simulation was applied to study the dynamic response of the surrounding rock on each side of the roadway in open-pit bench blasting.

### 3.1. Engineering Survey

**Figure 4:**
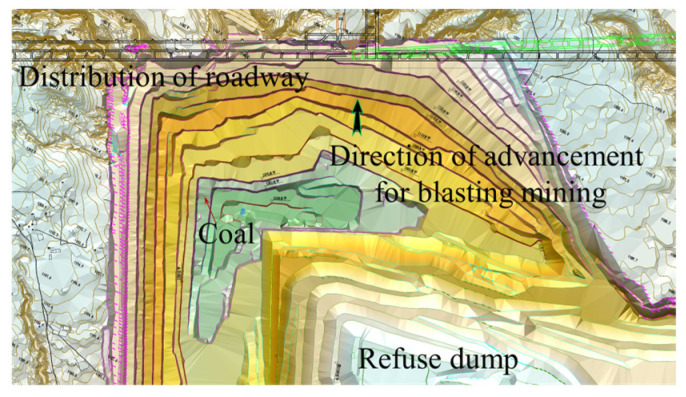
Relative position between open-pit cast coal mine and roadway.

**Figure 5:**
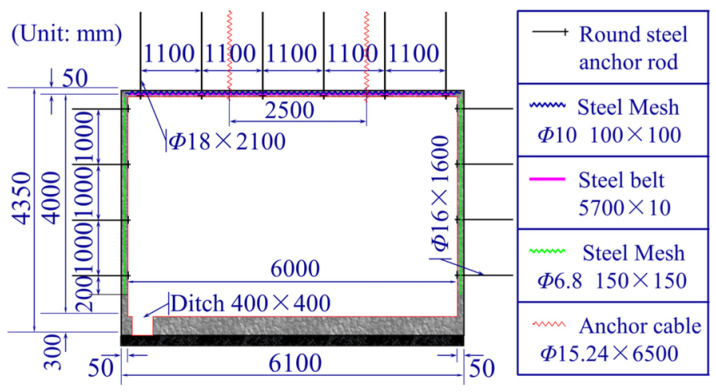
Roadway section and support parameters.

The Shangwan underground coal mine and Wujiata open-pit coal mine are located in Ordos, China. The air-return roadway of the Shangwan underground coal mine was located adjacent to the Wujiata open-pit coal mine and parallel to the northern boundary of the coal mine. The relative position between the open-pit mine and the roadway is shown in Figure 4; the cross-section of the roadway was rectangular, with an effective width of 6000 mm and a height of 4000 mm, and was supported by bolt-shotcrete. Specifically, the roof of the roadway was supported by the combination of round steel anchor stock and anchor cables, while the sides of the roadway were supported by anchor stock. The thickness of the shotcrete applied to the roadway surrounding rock was 50 mm, with a strength grade of C20. The thickness of the concrete on the roadway floor was 300 mm, with a strength grade of C30. The parameters of the roadway cross-section and the support parameters are shown in Figure 5.

### 3.2. Establishment of Finite Element (FE) Model

**Figure 6:**
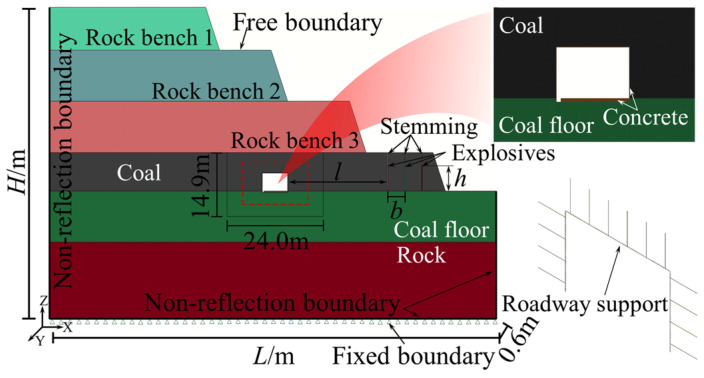
FE computational modeling of bench blasting (example figure, 30 m).

Based on the actual working conditions of bench blasting in an open-pit coal mine adjacent to the underground roadway, the explicit dynamics analysis software Ansys/Ls-dyna (2022) was applied. The quasi-three-dimensional FE computational models were developed for open-pit bench blasting with distances of 30~120 m from the roadway and with relative angles of 0°, 42°, and 90°, respectively. The schematic of the models is shown in Figure 6. According to the actual blasting parameters, the borehole diameter was 200 mm, the row spacing was 5.0 m, the charge length was 6.0 m, and the charge was a decoupled structure. The FE computational model parameters are listed in Table 1.

In the numerical simulation, the rock mass above the coal seam was set as the same type, and multiple layers were utilized for bench blasting, with each bench having identical material parameters. Therefore, the reflection and transmission effects of the stress wave within the rock mass above the coal seam were not considered. To address the coupling between the anchor stock and the units of the roadway surrounding rock, it was assumed that the anchor stock was fully bonded with the roadway surrounding rock. The coupling parameters are set by using the keyword *CONSTRAINED_LAGRANGE-IN_SOLID. A non-reflecting boundary condition was set up at the bottom and the left side of the numerical model to prevent the stress reflection on the calculation results.

Gravitational acceleration was applied in the vertical direction, with fixed boundary conditions imposed at the bottom of the model. The Arbitrary Lagrangian Eulerian (ALE) algorithm was selected between the rock and the explosive to avoid the loss of computational accuracy due to Lagrangian grid distortion during large deformation analysis.

#### 3.2.1. Explosive Model and State Equation

The explosive parameters were mainly set with reference to the actual blasting parameters. *MAT_HIGH_EXPLOSIVE_BURN was selected as the explosive material, and the *EOS_JWL state equation (Equation (30)) was used to describe the relationship between the relative volume V and the initial internal energy per elements volume $E_{0}$ under the explosion pressure [37].

(30) $$P=A\left(1-\frac{\omega }{R_{1}V}\right)e^{-R_{1}V}+B\left(1-\frac{\omega }{R_{2}V}\right)e^{-R_{2}V}+\frac{\omega E_{0}}{V_{0}}$$

where P is the explosive pressure, Pa; $V_{0}$ is the initial relative volume; $R_{1}$, $R_{2}$, and ω are the state constants of the equation; $E_{0}$ is the initial internal energy, J/m^3^; and A and B are the material constants.

The material parameters and equation of state parameters are listed in Table 2.

#### 3.2.2. Coal and Rock Materials

The *MAT_JOHNSON_HOLMQUIST_CONCRETE material model considers the strain rate effect and damage evolution mechanism of brittle materials under explosion and shock loading. It is the most commonly used material model for rocks, concrete, and other materials [38,39]. The expression is defined as:

(31) $$\sigma^{*}=[A(1-D)+Bp^{*N}][1+C\ln({\dot{\varepsilon }}^{*})]\;\sigma^{*}=\sigma /f_{c}^{\prime };\;p^{*}=p/f_{c}^{\prime };\;{\dot{\varepsilon }}^{*}=\dot{\varepsilon }/{\dot{\varepsilon }}_{0}\;D=\sum \left[(\Delta \varepsilon_{p}+\Delta \mu_{p})/\left(D_{1}{\left(p^{*}+T^{*}\right)}^{D_{2}}\right)\right];\;T^{*}=T/f_{c}^{\prime }$$

where $\sigma^{*}$ is the normalized equivalent stress; $p^{*}$ is the normalized pressure; ${\dot{\varepsilon }}^{*}$ is the dimensionless strain rate; $T^{*}$ is the normalized maximum tensile hydrostatic pressure; σ is the actual equivalent stress; p is the pressure; $f_{c}^{\prime }$ is the quasi-static uniaxial compressive strength; $\dot{\varepsilon }$ is the actual strain rate; ${\dot{\varepsilon }}_{0}$ is the reference strain rate; A is the cohesion parameter; B is the hydrostatic pressure parameter; N is the hydrostatic pressure index; C is the strain rate parameter; D is the damage variable, 0≤D≤1; $D_{1}$ and $D_{2}$ are the material damage constants; $\Delta \varepsilon_{P}$ is the equivalent plastic strain increment; $\Delta \mu_{P}$ is equivalent plastic volume strain increment; and T is the material maximum tensile hydrostatic pressure.

In this research, this material model was used to simulate coal, rock [39], and anchored concrete [38]. The relevant parameters are presented in Table 3.

#### 3.2.3. Anchor Stock Material

In blasting operation, the anchor material model should fully consider the strain rate effect of the material. The model *MAT-PLASTIC_KINEMATIC is a strain rate-dependent elastic–plastic material model with failure criteria; the relationship between the tensile strength of materials and strain rate, as well as the compressive yield strength and strain rate, was described using the Cowper Symonds model. The material parameters are shown in Table 4.

The parameters for the air materials and the stemming material in this paper are as given in [6].

### 3.3. Validation of the Numerical Model

The maximum quantity per delay, the distance between the blasting source and the measured point, blasting parameters, geological conditions, and additional factors influence the blast-induced ground vibration. According to safety regulations for blasting (GB6722-2014) [40], the empirical equation proposed by Sadovski was used to predict the peak particle velocity (PPV), as expressed in Equation (32).

(32) $$\mathrm{PPV}=K{\left(W^{1/3}/R\right)}^{\alpha }$$

where PPV is the peak particle velocity, cm/s; W the maximum quantity per delay of emulsion explosive, kg; R the distance between the blasting source and the measured point, m; and K and α are the geological correlation coefficient.

**Figure 7:**
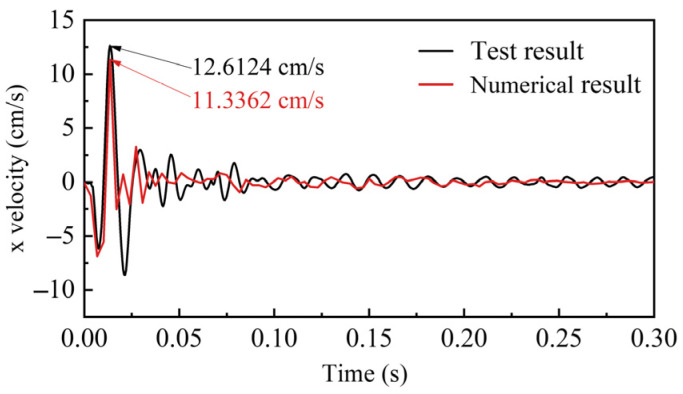
Errors between predicted and actual PPV values.

**Figure 8:**
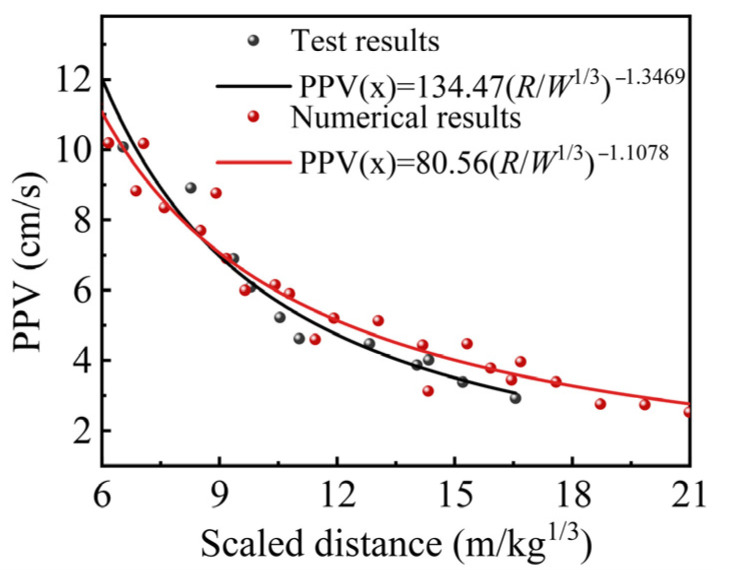
Numerical model validation.

To validate the accuracy and reliability of the model parameters, the results of on-site single-hole blasting and numerical simulation were compared. The velocity–time curves for both the experimental and simulated velocities are shown in Figure 7, while Figure 8 presents the test and simulated PPV fitted using Sadovski’s equation. The results confirm that the model parameters used in this paper can accurately simulate the stress distribution and vibration response of the surrounding rock in the roadway under the explosion.

## 4. Numerical Results

### 4.1. Analysis of the Cavity Effect on the Roadway Surrounding Rock

**Figure 9:**
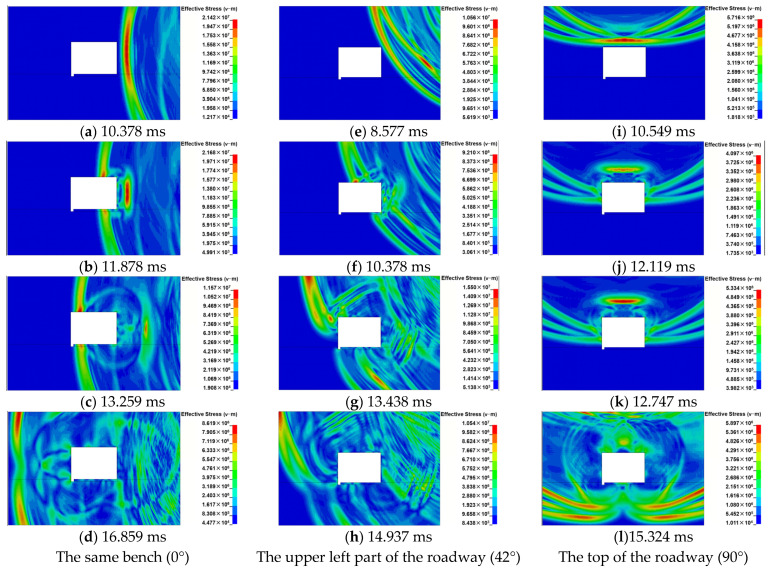
Stress wave propagation in roadway surrounding rock.

Numerical simulations of the dynamic disturbance on the roadway surrounding rock in open-pit bench blasting with different distances and bench heights were carried out. In order to investigate the influence of bench height on the cavity effect in open-pit bench blasting, the stress clouds of roadway surrounding rock in different blasting locations were produced and are shown in Figure 9.

In order to further analyze the stress wave propagation in the roadway surrounding rock, the blasting source at the right, upper left, and top of the roadway were simulated. The resulting stress maps are shown in Figure 9a–d, Figure 9e–h, and Figure 9i–l, respectively. It is concluded that the stress wave propagates to the free face of the roadway surrounding rock, and the stress wave is reflected at the free interface of the roadway due to the influence of the free face, with the unloading wave following the incident pressure loading wave and the surface-reflected stress wave to produce complex stress wave superposition action at the free interface of the roadway surrounding rock. Furthermore, the face-explosion side of the roadway surrounding rock is the main influence area under the repeated tensile and compressive effects of the stress wave. In the back-explosion side of the roadway, stress wave wave fronts move in opposite directions on both sides and converge in the middle of the back-explosion side. The stress wave effect was enhanced, but the dynamic response on the back blast side of the surrounding rock was smaller.

It is also deduced that the stress wave propagation direction is affected by the cavity roadway, and the stress wave propagates around the roadway to the undisturbed area. The disparity in the incidence angles of the stress wave wave front at each mass point of the roadway surrounding rock leads to the difference among the effects of the reflection and superposition stress waves at each mass point. Meanwhile, the effects cause different stress states at the mass points, indicating that the difference in stress state is related to the angle of the incident stress wave on the free face of the roadway surrounding rock. The difference in blast loading on each side of the rectangular roadway surrounding rock indicates the cavity effect of the roadway on the propagation of the blasting stress wave around the rectangular roadway.

It is seen in Figure 9 that when the location of the blasting source was changed from the same bench to the top of the roadway surrounding rock, the stress wave intensity reaching the roadway was reduced. Meanwhile, the relative angle of the stress wave wave front acting on the roadway was also changed. It led to a change in the reflected wave angle on the roadway surrounding rock and the regional location of the reflection and superposition effect of stress wave on the free face of the roadway rock, causing significant disparities in the stress distribution with the roadway surrounding rock under different levels of bench blasting. It is demonstrated that the relative angle (θ) is one of the critical factors affecting the stress evolution and dynamic damage of the roadway surrounding rock.

### 4.2. Particle Velocity Distribution on the Rectangular Roadway Surrounding Rock

**Figure 10:**
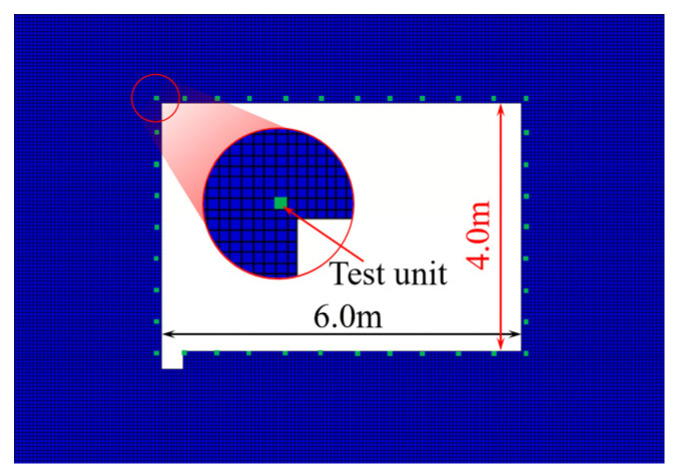
Test unit distribution of particle velocity of roadway enclosure rock.

**Figure 11:**
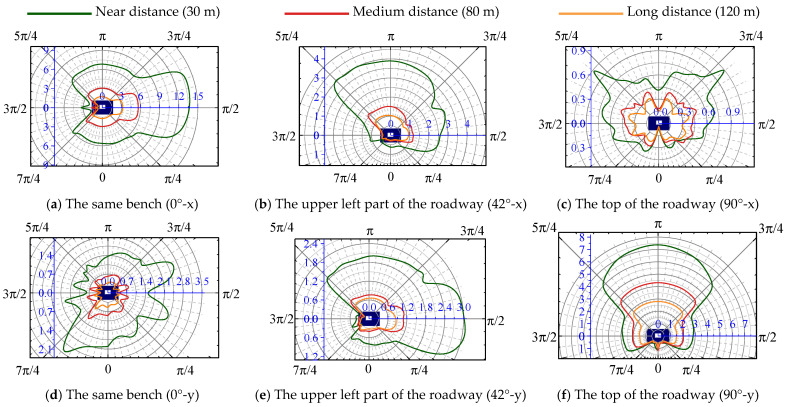
Changing the law of the PPV of roadway surrounding rock under the effect of different bench blasting.

Equally spaced units along the contour of the roadway surrounding rock were selected to monitor the PPV. The selected units are shown in Figure 10. To investigate the radial and tangential vibration distribution law, different distances from the blasting source to the selected units and benches were selected. The variation curves of the surrounding rock’s PPV are presented in Figure 11.

Figure 11 shows that the PPV of the profile surface of the roadway surrounding rock is significantly different. The PPV of the face-explosion side affected by the explosion was the largest. Compared with the PPV of the face-explosion side, the PPV of the back-explosion side was significantly weak. However, the seismic wave converged in the middle area of the back-explosion side, and the vibration strength of the surrounding rock appeared to be a sudden leap.

Since $\sigma_{I_{P}}=z_{P}v_{I_{P}}$, for the same rock properties, the particle velocity of the surrounding rock is proportional to the dynamic stress. By the effect of roadway cavity, roadway rock free surface, and blasting seismic wave propagation and attenuation, the relative angle of the stress wave incident on the free face of the roadway surrounding rock mass is different, leading to varying particle velocities of each mass point of the roadway surrounding rock caused by the incident, reflection, and superposition effects of the stress wave. The energy of the blasting stress wave is rapidly attenuated during propagation. Therefore, the PPV of the roadway surrounding rock on each side is significantly different. This reflects the significant correlation between the PPV of the roadway surrounding rock and the incident angle of the stress wave.

The comparison of the PPV of the roadway surrounding rock under different levels of bench blasting revealed that the blasting position at the same bench of the roadway had the greatest impact on the PPV of the roadway surrounding rock, followed by top blasting, whereas side blasting resulted in the smallest PPV of the surrounding rock.

As the stress wave propagated from the rock medium to the coal medium, an unloading effect occurred at the stratified interface of rock, leading to further attenuation of the stress wave wave front. Additionally, the unloading intensity of the reflected and transmitted waves was influenced by the incident angle of the stress wave at the stratified interface. Above all, it indicated that the change in relative angle (*θ*) led to different stress intensities acting directly on the surrounding rock of the roadway, resulting in disparities in the PPV of the roadway surrounding rock. It reflected that the relative angle (*θ*) was a critical factor governing the vibration intensity of the surrounding rock on each side of the roadway.

### 4.3. Influence of Distance on the Cavity Effect

**Figure 12:**
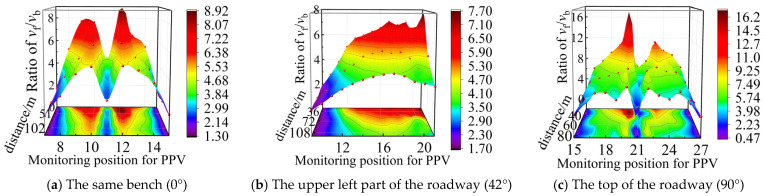
Ratio of the PPV between the face-explosion side and the back-explosion side of the roadway.

Figure 12a shows the maximum ratio of the PPV between the face-explosion side and the back-explosion side of the roadway as 5.06 when the distance from the blasting source to the roadway was 120 m. When the distance from the blasting source to the roadway was gradually reduced to 36 m, the ratio of the PPV was progressively increased to 8.13, and the difference in the PPV of the roadway surrounding rock was relatively significant. As the distance decreases, the cavity effect promotes a considerable increase in the ratio of the PPV of the roadway surrounding rock on each side of the roadway. From Figure 12b,c, the same conclusion is obtained for the cavity effect under the bench blasting at different locations.

In summary, the cavity effect changes the stress wave propagation in the roadway surrounding rock. The change in the relative angle (*θ*) between the blasting source and the roadway promotes an angle change in the stress wave wave front acting on the roadway surrounding rock, causing the angle of the blasting stress wave incident on the free face of the roadway surrounding rock to be different and resulting in differences in the dynamic response of the roadway surrounding rock. With the decrease in the distance from the blasting source to the roadway surrounding rock, the difference in particle velocities of the surrounding rock on each side was more pronounced.

It is shown that the relative angle (*θ*) significantly influences the dynamic response and stress distribution in the surrounding rock on each side. The numerical results are consistent with the theoretical results. Therefore, considering *θ* as a major factor that affects the vibration of surrounding rock in open-pit blasting is imperative.

## 5. Prediction of PPV of Surrounding Rock

### 5.1. Monitored Point of PPV

**Figure 13:**
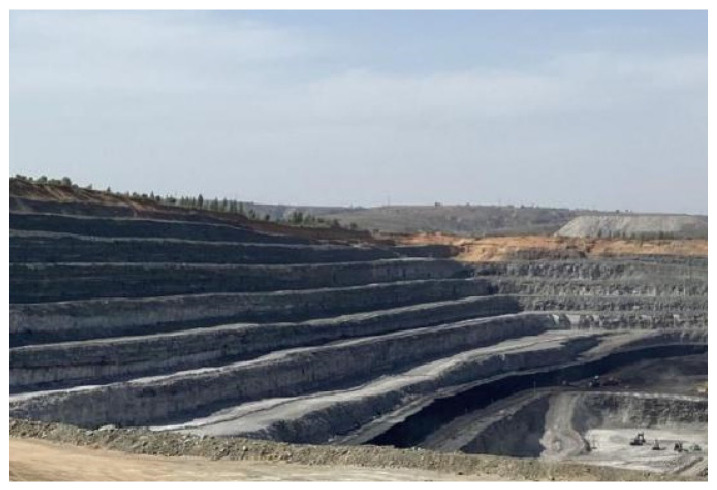
Open-pit coal mine site.

**Figure 14:**
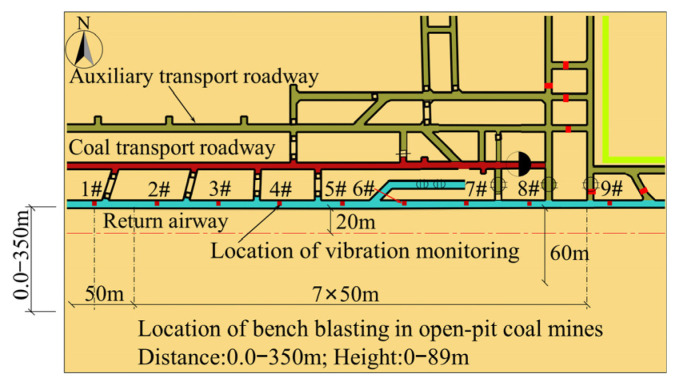
Location of monitored points of the roadway (localized).

**Figure 15:**
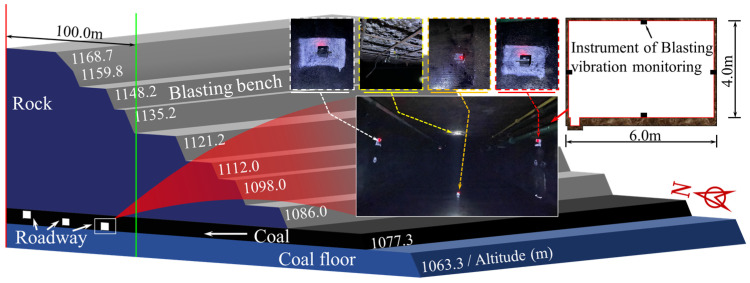
Schematic diagram of on-site installation of roadway surrounding rock vibration monitoring instruments.

The vibration velocity of the surrounding rock in the neighboring underground roadway caused by bench blasting operations in the Wujiata open-pit coal mine was monitored to predict the PPV of the surrounding rock. The monitoring site was in the air-return roadway of the Shangwan underground coal mine adjacent to the open-pit coal mine. The Wujiata open-pit coal mine is shown in Figure 13, while the relative locations of the blast test site and the monitoring site are depicted in Figure 14. Additionally, Figure 15 illustrates the vibration monitoring in the roadway surrounding rock of the Shangwan underground coal mine.

In open-pit bench blasting, the double-row or multi-row borehole was detonated hole by hole. The blasting parameters were as follows: borehole depth of 11.0 m, ultra-deep blasting of 1.0 m, borehole diameter of 200 mm, borehole spacing of 6.0 m, row spacing of 5.0 m, and stemming of 5.0 m. A mixed ammonium nitrate fuel oil (ANFO) explosive with a density of 1.1 g/cm^3^ and a detonation velocity of 3600 m/s was used.

**Figure 16:**
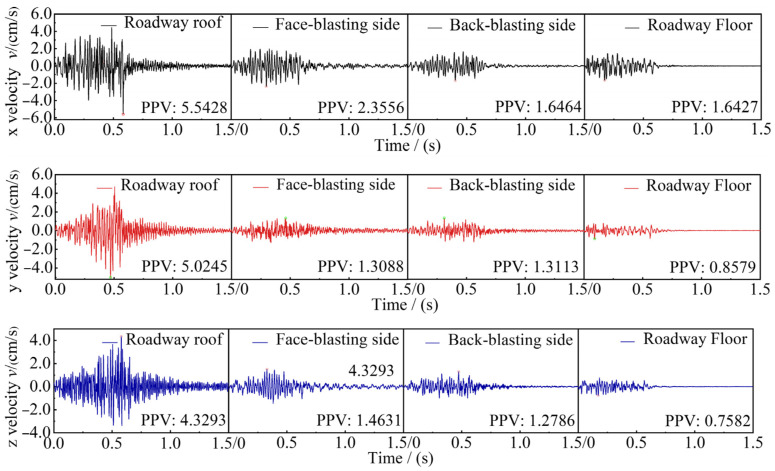
Time-dependent curve of particle velocity.

CBSD-VM-M01 network vibrometers were employed to monitor the vibration velocity in the roadway surrounding rock. Combined with the distribution location of the open-pit bench blasting area, the PPVs in the X, Y, and Z orthogonal directions on the roof, sidewalls, and the floor on the surrounding rock of the neighboring coal roadway were monitored, respectively. Due to space limitations, only a typical set of measured vibration signals of the roadway surrounding rock is shown in Figure 16. The vibration monitoring results of the roadway surrounding rock are shown in Table 5.

### 5.2. Regression Analysis of Peak Particle Velocity

Theoretical analysis and numerical simulation confirmed that the relative position of open-pit bench blasting and the roadway is one of the critical factors influencing the stress evolution and distribution of the PPV on the roadway surrounding rock. The change in the relative angle between the bench blasting area and the roadway causes significant variation in stress reflection and superposition effects of the roadway surrounding rock and changes the main influence area of the roadway surrounding rock under the action of open-pit bench blasting vibration. Therefore, the relative angle (*θ*) was introduced into the traditional Sadovski equation to more accurately evaluate the impact of open-pit bench blasting vibrations on roadway surrounding rock stability, obtain the applicable prediction equation of PPV to the roadway surrounding rock, and improve the PPV prediction accuracy.

#### 5.2.1. Developing Empirical Equation for Predicting PPV Considering the Relative Angle

The relative angle affects the vibration distribution trend in the roadway rock under open-pit bench blasting (*θ*). The main factors affecting the PPV of the roadway surrounding rock were considered, such as the maximum quantity per delay *W*, the charge radius *r*, the distance between the blasting source and the monitored point *L*, the vertical height from the blasting source to the roadway *H*, the horizontal distance between the blasting source and the roadway *l*, and the longitudinal wave velocity *C*_p_. The mass [*M*], length [*L*], and time [*T*] were taken as the primary dimension variables; *W*, *L*, and *C*_p_ were independent variables; the PPV was the dependent variable; and similar standard equations were obtained via magnitude analysis, expressed as Equations (33) and (34).

(33) $$\pi =\phi (\pi_{1},\pi_{2},\pi_{3})$$

(34) $$PPV=C_{p}\phi (\frac{H}{L},\frac{l}{L},\frac{r}{L})$$

According to the dimension analysis, $\pi_{1}\times \pi_{2}$ is obtained as $\pi_{12}=\pi_{1}\times \pi_{2}=Hl/L^{2}$. If the explosive type remains unchanged, a cubic power function relationship exists between the maximum quantity per delay and the charge radius [40], i.e., $r≅\sqrt[{3}]{W}$.

Then, Equation (32) can be rewritten as Equation (35).

(35) $$PPV{=C}_{p}\phi (\frac{Hl}{L^{2}},\frac{\sqrt[{3}]{W}}{L})$$

Since *H* and *l* are the vertical and horizontal components of the distance between the blasting source and the monitored point, respectively, *L* is the relation equation, and $L=\sqrt{H^{2}+l^{2}}$ exists. Thus, Equation (35) can be rewritten as Equation (36).

(36) $$\begin{array}{ll} PPV & =K{\left[\sin{\left(\theta \right)}^{2}-\sin{\left(\theta \right)}^{4}\right]}^{\beta }{\left(\frac{\sqrt[{3}]{W}}{R}\right)}^{\alpha }\\ & =K{\left[\frac{\sin{\left(2\theta \right)}^{2}}{4}\right]}^{\beta }{\left(\frac{\sqrt[{3}]{W}}{R}\right)}^{\alpha } \end{array}$$

#### 5.2.2. Validation of the Improved Empirical Equation

To validate the accuracy and applicability of the improved empirical equation for predicting the PPV of the roadway surrounding rock, vibration monitoring data from the roadway surrounding rock were utilized, as shown in Table 3. Equations (32) and (36) were employed for data curve fitting analysis using MATLAB (2021). The root mean square error (RSME), coefficient of determination ($R^{2}$), and modified coefficient of determination ($A\text{-}R^{2}$) were obtained. The corresponding equations are expressed as Equations (37)–(39), respectively. The smaller the RSME and $R^{2}$, the closer the $A\text{-}R^{2}$ to 1, indicating that the independent variable can respond better to the dependent variable and the data points fit better.

(37) $$RMSE=\sqrt{\frac{1}{n}\sum_{i=1}^{n}w_{i}{\left(y_{i}-y_{i}^{\prime }\right)}^{2}}$$

(38) $$R^{2}=1-\frac{\sum_{i=1}^{n}w_{i}{\left(y_{i}-y_{i}^{\prime }\right)}^{2}}{\sum_{i=1}^{n}w_{i}{\left(y_{i}-{\hat{y}}_{i}\right)}^{2}}$$

(39) $$A\text{-}R^{2}=1-\frac{\left(1-R^{2})(n-1\right)}{n-p-1}$$

where $w_{i}$ is the data sample weight; $y_{i}$ is the original data; $y_{i}^{\prime }$ is the predicted data; ${\hat{y}}_{i}$ is the original data mean; *n* is the number of samples; and *p* is the number of features.

(1) Fitting analysis of Sadovski equation

**Figure 17:**
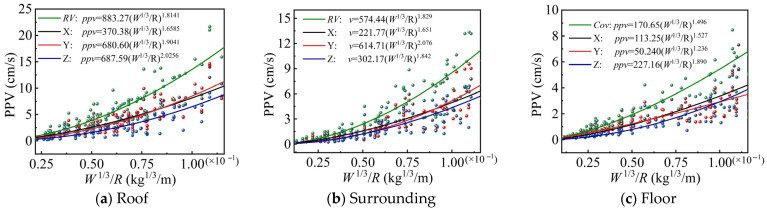
Curve fitted using Sadovski equation.

The monitoring data of the roadway surrounding rock was applied for curve fitting using Equation (32). The fitting curve of the PPV of the roadway surrounding rock was obtained and is shown in Figure 17. The results of the data fitting are presented in Table 6.

Figure 17 and Table 6 indicate that the fitted PPV values of the surrounding rock on each side of the roadway exhibit a clear nonlinear decreasing trend. The data of PPV were greatly dispersed, and the correlation coefficients $R^{2}$ and $A\text{-}R^{2}$ were relatively low, so the RSME value was large in regression analysis. Therefore, it can be concluded that the Sadovski equation has certain limitations in accurately predicting the PPV of the surrounding rock on each side of the roadway.

(2) Fitting analysis of the improved equation

**Figure 18:**
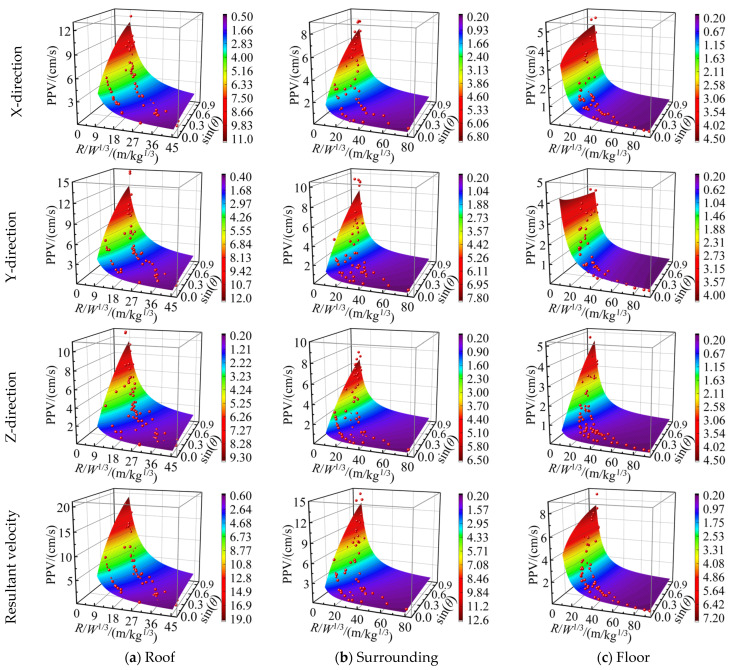
Fitted surface introducing the relative angle (*θ*) improved the Sadovski equation.

The improved Equation (36) considering the relative angle (*θ*) was applied to the regression analysis. The fitting surface of the PPV on the roadway surrounding rock is shown in Figure 18, and the fitting results are listed in Table 7.

Figure 18 and Table 7 show that the PPV of the surrounding rock on each side of the roadway exhibits a nonlinear declining trend with the variation in the scale distance and the relative angle (*θ*). Meanwhile, the scatter of PPV data matches well with the fitted surface of the improved equation for predicting the PPV by considering the relative angle (*θ*), signifying the importance of the relative angle (*θ*) in predicting the PPV.

Compared with the regression analysis results of Sadovski’s empirical equation and the modified equation in Table 6 and Table 7, the modified equation for predicting the PPV of the roadway rock has smaller RMSE values in the regression analysis of the monitoring data of the roadway roof and the right side of the surrounding rock. The fitting values of the $R^{2}$ and $A\text{-}R^{2}$ parameters were considerably improved. The prediction results are found to be better than those predicted using the traditional Sadovski empirical equation, corroborating the effectiveness of the relative angle (*θ*) to improve the equation for predicting the PPV of the roadway surrounding rock. The numerical simulation results indicate that the relative angle (*θ*) of the open-pit bench blasting area and the roadway have little influence on the PPV of the roadway floor. Therefore, the error assessment parameters such as RMSE, $R^{2}$, and $A\text{-}R^{2}$ reflect that the prediction accuracy has not improved. The results of the fitting analysis and the conclusions obtained from the numerical simulation are compatible and corroborated. To some extent, the accuracy and reliability of the improved Equation (36) for predicting the PPV of the roadway surrounding rock are verified.

The modified equation incorporating the relative angle (*θ*) for predicting the PPV of the roadway surrounding rock can effectively predict the PPV compared to the traditional Sadovski equation. However, under frequent open-pit bench blasting operations, it is suggested that vibration monitoring be strengthened, and Equation (36) must be improved for accurate prediction of PPV.

## 6. Conclusions

In this paper, the dynamic response and stability of the adjacent rectangular roadway in open-pit mining were investigated, and the blast-induced vibration of the adjacent rectangular roadway was measured. The vibration equations of the roadway were derived based on the stress wave propagation theory and the wave-front momentum conservation theorem. Numerical simulation was conducted to analyze the stress distribution and dynamic response of the roadway for varying distances and bench heights. The following conclusions were drawn from the results:

(1) A mathematical model of underground roadway disturbed by open-pit bench blasting was established through theoretical analysis. The vibration equation of the rectangular roadway under the influence of blasting seismic waves and surface-reflected stress waves was developed using the theory of waveform propagation and conservation of wave-front momentum.
(2) The numerical simulation results show that with the influence of the effect of the free face of the roadway surrounding rock, there is a significant difference in the degree of PPV response to the blasting power of each measurement point on the free face of the surrounding rock on each side of the roadway. The severity of this difference varies with the change in the relative angle (*θ*), which has the most pronounced effect on the face blast side of the surrounding rock.
(3) The effect of the roadway cavity enhances the dynamic response on each side of the surrounding rock, contributing to the difference in the PPV of each side of the roadway as the distance from the blasting center to the monitoring point decreases. The difference in the dynamic response of the roadway is especially significant.
(4) Based on Sadovski’s empirical equation, the modified equation for predicting the PPV was established considering the relative angle (*θ*). The $R^{2}$ obtained from the regression analysis confirms the better prediction accuracy of the modified equation. The improvement in prediction accuracy of the roadway PPV is related to the degree of dynamic response on the roadway with blasting load.

## Figures and Tables

**Table 1:** Parameters of FE computational model in bench blasting.

Model Number	Model Dimension		Distance	Relative Angle	Blast Hole Parameter			Total Number of Benches
	*L* (m)	*H* (m)	*l* (m)	*θ* (°)	*Φ* (m)	*b* (m)	*h* (m)	
B-120m-0	201.0	108	120	0	0.20	4.0	6.0	9
B-120m-42	204.0	139	120	42	0.20	4.0	6.0	12
B-120m-90	117.0	150	90	90	0.20	4.0	6.0	13
B-80m-0	151.0	85	80	0	0.20	4.0	6.0	7
B-80m-42	151.0	108	80	42	0.20	4.0	6.0	9
B-80m-90	106.0	120	60	90	0.20	4.0	6.0	11
B-30m-0	101.0	67	30	0	0.20	4.0	6.0	6
B-30m-42	126.0	67	30	42	0.20	4.0	6.0	6
B-30m-90	87.0	90	30	90	0.20	4.0	6.0	8

**Table 2:** Explosive material parameters and *EOS_JWL parameters.

RO	*D*	PCJ	BETA	*K*	*G*	SIGY	*A*	*B*	*R*_1_	*R*_2_	*ω*	*E*_0_	*V*_0_
(kg/m^3^)	(m/s)	(GPa)				(Pa)	(GPa)	(GPa)				(J/m^3^)	
1020	3600	5.15	0	0	0	0	49.46	1.89	3.9077	1.118	0.33	2.668 × 10^9^	1

**Table 3:** Parameters of coal, rock, and anchored concrete.

Parameters	Coal	Concrete	Sandstone	Parameters	Coal	Concrete	Sandstone
ρ (kg/m^3^)	1248	2660	2010	PC (MPa)	6.210	16.00	8.267
G (GPa)	1.135	14.86	4.576	UC	0.0027	0.001	0.00342
A	0.60	0.79	0.61	PL (GPa)	0.8	0.8	0.8
B	1.769	1.6	1.92	UL	0.069	0.1	0.069
C	0.00186	0.007	0.00108	D1	0.04	0.04	0.04
N	0.7515	0.61	0.80	D2	1.0	1.0	1.0
$f_{c}$ (MPa)	18.63	48.0	24.8	K1 (GPa)	43	85	43
T (MPa)	1.64	4	2.51	K2 (GPa)	−257	−171	−257
EFMIN	0.01	0.01	0.01	K3 (GPa)	596	208	596

**Table 4:** Parameters of anchor stock material.

RO (kg/m^3^)	*E* (GPa)	PR	SIGY (MPa)	*E*_tan_ (MPa)	*C* (s^−1^)	*P*
7800	209	0.3	345	2100	300	5

**Table 5:** Blasting parameters and roadway surrounding rock vibration monitoring results (partial).

Group	Roof					Surrounding					Floor				
	*θ*	*R*/*W*^1/3^	PPV/(cm/s)			*θ*	*R*/*W*^1/3^	PPV/(cm/s)			*θ*	*R*/*W*^1/3^	PPV/(cm/s)		
	(°)	(m/kg^1/3^)	X	Y	Z	(°)	(m/kg^1/3^)	X	Y	Z	/(°)	(m/kg^1/3^)	X	Y	Z
1	10.14	15.97	2.68	1.77	1.11	9.13	17.62	0.99	0.88	0.79	8.23	28.27	0.83	0.88	0.36
2	9.86	16.34	2.52	1.85	1.12	8.72	19.74	1.22	0.52	0.70	7.12	33.41	0.82	0.62	0.34
3	7.38	19.69	1.62	2.28	1.40	6.70	21.56	0.81	1.04	0.59	5.90	39.38	0.45	0.50	0.26
4	9.13	17.62	2.56	1.56	0.70	5.50	25.49	0.74	0.71	0.52	5.50	43.22	0.40	0.39	0.27
5	7.38	19.69	1.52	1.89	0.70	4.02	41.32	0.50	0.39	0.47	7.27	61.40	0.27	0.31	0.23
6	5.48	29.14	0.72	0.84	0.44	5.45	29.08	0.36	0.37	0.28	6.90	70.18	0.17	0.22	0.19
7	5.48	29.14	0.74	0.83	0.34	4.86	32.63	0.48	0.48	0.24	9.02	55.11	0.22	0.26	0.13
8	14.63	16.08	4.95	4.73	2.33	7.27	61.40	0.46	0.29	0.39	5.81	78.39	0.18	0.13	0.13
9	14.07	15.46	4.56	4.46	2.02	5.81	78.39	0.33	0.19	0.22	5.40	87.02	0.12	0.12	0.12
10	13.38	11.90	4.11	4.53	1.37	11.43	14.18	2.83	1.97	1.41	5.01	92.02	0.11	0.13	0.11
11	12.48	12.88	3.81	2.26	1.23	14.12	10.90	2.11	2.01	2.11	14.14	34.30	1.28	0.62	0.82
12	12.48	12.88	2.84	2.88	1.40	14.07	10.46	2.63	1.99	1.25	10.55	22.62	1.39	1.60	0.74
13	11.24	14.17	3.19	2.04	1.66	13.60	11.76	1.80	1.85	1.91	10.31	22.61	1.23	1.68	1.03
14	11.37	14.22	2.94	2.56	1.04	13.38	11.90	2.00	1.93	1.44	13.83	34.25	1.11	0.88	0.52
15	12.37	23.82	0.91	0.78	0.45	10.14	15.97	2.08	1.69	1.24	10.17	23.47	1.18	1.35	0.42
16	12.48	35.95	0.43	0.68	0.37	13.47	45.55	0.71	1.00	0.39	13.65	36.46	0.80	0.71	0.36
17	12.13	35.90	0.49	0.55	0.23	12.25	25.82	0.42	0.81	0.50	13.93	33.24	1.11	0.88	0.41
18	13.22	44.47	0.23	0.25	0.18	13.42	54.36	0.72	0.41	0.25	14.35	37.88	0.91	0.60	0.48
19	15.09	9.54	5.42	5.79	3.63	10.65	29.22	0.47	0.49	0.32	10.98	29.19	0.79	0.88	0.70
20	16.28	33.91	1.72	1.89	1.05	15.09	9.54	3.02	4.07	2.53	12.52	25.67	1.09	0.78	0.43
21	15.69	34.91	1.10	1.27	0.99	18.70	16.94	2.52	1.48	1.83	10.43	29.14	0.72	0.83	0.64
22	17.91	23.80	1.25	0.85	0.98	17.77	34.97	0.76	1.15	0.60	11.17	41.47	0.69	0.63	0.45
23	16.11	33.88	1.34	1.06	0.49	16.47	32.95	1.21	0.66	0.64	12.21	27.43	0.53	0.64	0.47
24	16.47	32.95	1.22	0.39	0.45	15.69	34.91	0.92	0.73	0.37	10.04	47.92	0.52	0.48	0.26
25	15.89	34.41	1.00	0.69	0.49	19.60	23.07	0.78	1.10	0.81	11.00	31.21	0.42	0.48	0.35
26	15.03	38.16	0.54	0.30	0.30	19.02	34.93	0.87	0.71	0.55	13.47	42.04	0.49	0.39	0.36
27	19.08	34.79	0.40	0.33	0.26	15.66	37.78	0.61	0.56	0.28	10.80	45.05	0.51	0.27	0.40
28	19.08	43.72	0.34	0.30	0.35	15.03	38.16	0.63	0.30	0.29	13.42	54.36	0.31	0.24	0.21
29	21.41	25.93	2.96	3.87	1.92	20.39	16.65	1.93	2.11	2.47	18.70	16.94	1.87	1.32	0.97
30	24.01	12.97	4.02	3.62	1.55	22.84	27.09	1.09	1.01	1.27	19.22	18.16	1.44	1.75	0.98

Note: The X, Y, and Z directions represent the horizontal radial, horizontal tangential, and vertical directions, respectively.

**Table 6:** Parameter fitting results of Sadovski equation.

Fitted Parameters	Roof				Surrounding				Floor			
	X	Y	Z	RV	X	Y	Z	RV	X	Y	Z	RV
*k*	370.38	680.60	687.59	883.27	221.77	614.71	302.17	574.44	113.25	50.240	227.16	170.65
*n*	1.6584	1.9041	2.0256	1.8141	1.6513	2.0758	1.8415	1.8290	1.5272	1.2363	1.8899	1.4960
*RMSE*	1.2701	1.5132	1.2621	2.0463	1.0406	1.2472	1.0424	1.6421	0.7534	1.2965	0.7295	1.0165
*R* ^2^	0.7855	0.7469	0.7100	0.8005	0.7444	0.7120	0.7077	0.7857	0.7352	0.7314	0.7183	0.8009
*A*-*R*^2^	0.7830	0.7439	0.7067	0.7982	0.7409	0.7080	0.7037	0.7828	0.7314	0.7279	0.7146	0.7982

**Table 7:** Parameter fitting results of the improved equation.

Fitted Parameters	Roof				Surrounding				Floor			
	X	Y	Z	RV	X	Y	Z	RV	X	Y	Z	RV
*k*	295.91	524.31	420.07	664.19	139.60	335.34	184.96	339.94	99.174	52.168	177.92	138.65
−*α*	1.3408	1.5339	1.4763	1.4342	1.1756	1.4566	1.2704	1.2870	1.3998	1.284	1.3988	1.3023
*β*	0.2355	0.2765	0.3682	0.2760	0.2872	0.3777	0.3783	0.3227	0.078	0.0225	0.4356	0.1162
*RMSE*	0.9457	1.1763	0.8881	1.3418	0.8040	0.9789	0.7403	1.1511	0.7505	0.6311	0.6955	0.9916
*R* ^2^	0.8811	0.8471	0.8558	0.9142	0.8474	0.8225	0.8520	0.8983	0.7372	0.7317	0.7576	0.8056
*A*-*R*^2^	0.8783	0.8485	0.8525	0.9122	0.8432	0.8176	0.8479	0.8954	0.7302	0.7246	0.7512	0.8004

## Data Availability

The data presented in this study are available on request from the corresponding author due to the data also being utilized in the preparation of a dissertation, making them unavailable for public disclosure.
